# Evaluating the Role of Anti-PEG Antibodies in Hypersensitivity to PEG-Asparaginase Through Clinically Relevant Cut-Points Defined by ELISA and Surface Plasmon Resonance

**DOI:** 10.3390/pharmaceutics18101196

**Published:** 2026-09-22

**Authors:** Camilla Rossi, Alessia Porta, Beatrice Rocutto, Francesco Medici, Marta Cancelliere, Cristina Matteo, Antonella Colombini, Laura Rachele Bettini, Francesca Romani, Davide Colombo, Marten Beeg, Paolo Ubezio, Massimo Zucchetti, Carmelo Rizzari, Marco Gobbi

**Affiliations:** 1 Istituto di Ricerche Farmacologiche Mario Negri IRCCS, 20156 Milan, Italy; camilla.rossi@marionegri.it (C.R.); alessia.porta@marionegri.it (A.P.); rocuttobeatrice@gmail.com (B.R.); marta.cancelliere@marionegri.it (M.C.); cristina.matteo@marionegri.it (C.M.); marten.beeg@marionegri.it (M.B.); paolo.ubezio@marionegri.it (P.U.); massimo.zucchetti@marionegri.it (M.Z.); 2 Scuola di Medicina e Chirurgia, Università degli Studi di Milano-Bicocca, 20900 Monza, Italy; francescomedici95@gmail.com (F.M.); f.romani2@campus.unimib.it (F.R.); d.colombo@campus.unimib.it (D.C.); 3 Unità di Ricerca Clinica e Fase 1 Pediatria, Fondazione IRCCS San Gerardo dei Tintori, 20900 Monza, Italy; anto.colomb@gmail.com (A.C.); carmelo.rizzari@irccs-sangerardo.it (C.R.); 4 Clinica Pediatrica, Fondazione IRCCS San Gerardo dei Tintori, 20900 Monza, Italy

**Keywords:** PEG-asparaginase, acute lymphoblastic leukemia, anti-PEG antibodies, ELISA, surface plasmon resonance assay

## Abstract

**Background/Objectives**: Conjugation of polyethylene glycol (PEG) to L-asparaginase (ASP) reduces immunogenicity and prolongs the half-life of this biological drug used to treat acute lymphoblastic leukemia (ALL). However, some patients develop anti-drug antibodies (anti-ASP and anti-PEG), which may impair efficacy through neutralization or faster elimination and may cause hypersensitivity reactions. Their clinical impact remains debated, partly due to variability in analytical methods and cut-points often defined by statistical rather than clinical outcomes. **Methods**: To address this issue, we used two assays to measure anti-PEG antibodies in 27 sera from 25 children with ALL who showed reactions to PEG-ASP—either clinical allergy with reduced ASP activity (n = 18) or allergic-like reactions without major activity loss (n = 9)—and in 25 sera from 19 children with ALL but without clinically relevant reactions. Sera from 16 healthy children were used as controls. This design enabled assessment of sensitivity and specificity using clinically relevant cut-points. **Results**: Anti-PEG IgG (but not IgM), measured by ELISA, correlated with reduced drug activity, showing 83% sensitivity and 91% specificity. We also developed a surface plasmon resonance (SPR) assay, capable of detecting simultaneously both anti-PEG and anti-ASP antibodies and analyzing binding kinetics. ROC analysis of SPR data showed 78% sensitivity and 94% specificity. **Conclusions**: Overall, these findings highlight the importance of clinically defined cut-points, support a key role of anti-PEG antibodies in hypersensitivity with reduced drug activity, and suggest that measuring anti-PEG antibodies may help guide therapeutic decisions.

## 1. Introduction

Acute lymphoblastic leukemia (ALL) is the most common form of cancer in children [1]. Thanks to scientific research and current chemotherapy protocols, it is nowadays considered a disease with a very good prognosis, with over 90% of children surviving five years after diagnosis [2].

One of the most effective drugs for ALL is L-asparaginase (ASP), an enzyme able to deprive leukemic cells of asparagine, an amino acid essential for their growth. A main problem of ASP—like other recombinant biologicals obtained in bacteria—is the occurrence of hypersensitivity reactions, determined by anti-drug antibodies that can affect the efficacy and safety of the drug, neutralizing it and accelerating its clearance with or without allergy symptoms (the latter indicated as silent inactivation) [3,4]. These hypersensitivity reactions occur in up to 25–45% of cases with the native form of the enzyme [5]. The current first-line therapy protocols use PEG-ASP (Oncaspar^®^), a derivative form of the native *Escherichia coli* L-ASP covalently conjugated to polyethylene glycol (PEG) polymers. In fact, PEG is a very weak antigen [6] and is often exploited to mask the immunogenic epitopes of biological drugs, thus extending their half-lives. In the case of PEG-ASP, approximately 69–82 molecules of PEG (5 kDa each) are linked per enzyme molecule. Despite a decreased immunogenic potential, still 3.3–13.5% of patients treated with PEG-ASP show hypersensitivity reactions, most of them associated with anti-PEG antibodies [7,8,9], either induced by the treatment [7,8,10] or “pre-existing” [11,12]. The latter refer to antibodies present in individuals who have never undergone treatment with PEGylated biological drugs but, possibly, induced by the exposure to the PEG present in many daily use products, e.g., cosmetics and pharmaceutical industry products including surfactants, dispersing agents or medications (e.g., PEG-based osmotic laxatives). The prevalence of “pre-existing” anti-PEG antibodies reported in the literature is highly variable (4–72%) [10,11,13,14,15,16,17,18], most probably due to the different populations tested or to the period of sampling [19] or to the different detection tests associated with different classification thresholds [20].

The impact of the treatment-induced or pre-existing anti-PEG antibodies on the safety and efficacy of PEGylated drugs is still controversial, possibly because it depends on the quality and concentration of these antibodies and the cut-points of analytical methods are often defined by statistical approaches, rather than clinically validated. With statistical approaches, identifying samples significantly exceeding the background, it may happen that very sensitive methods such as enzyme-linked immunosorbent assay (ELISA) identify as “positive” samples with very low-and clinically-insignificant levels of anti-PEG antibodies, resulting in poor specificity.

The aim of the present work was therefore to clarify whether the measurement of anti-PEG antibodies could be useful to predict, with adequate sensitivity and specificity, the immunological response to PEG-ASP. Such information might allow clinicians to optimize and even personalize the treatment, e.g., to predict patients at risk of clinical allergy due to pre-existing anti-PEG antibodies or for a rational drug switch in the case of hypersensitivity reaction to the PEGylated drug.

A variety of methods have been reported to detect anti-PEG antibodies, such as passive hemagglutination [13,21], flow cytometry [15,17], Western blot [22], surface plasmon resonance (SPR) [23,24] and enzyme-linked immunosorbent assay (ELISA) [7,10,16,17,18,25], although the latter has been the most widely used. Both ELISA and SPR employ PEG-derivatives, coated on plate wells or on SPR sensor chips, to capture the anti-PEG antibodies present in serum samples. The quantification of the captured antibodies requires a number of additional steps in the ELISA assay (i.e., washings and incubations), whereas no additional steps are required with the SPR assay, which allows a direct, real-time and much more rapid detection of the captured antibodies [26]. Some SPR instruments allow the immobilization of different ligands on parallel strips of the same sensor surface, to capture and detect simultaneously different analytes present in the same solution, in a single run and on a single chip. Thus, one important advantage of SPR is the possibility to detect simultaneously, in the same serum sample, a biological drug and the corresponding anti-drug antibodies [26,27] or anti-drug and anti-PEG antibodies induced after administration of a PEGylated biological drug. Another advantage of SPR in comparison with ELISA is the possibility to appreciate the association and dissociation rates of the binding of serum anti-PEG antibodies with immobilized PEG [27], providing important indications regarding their binding properties.

In the present study, we analyzed serum samples from 17 pediatric ALL patients showing hypersensitivity reactions to PEG-ASP, 8 patients showing allergic-like reactions (which do not affect ASP activity, e.g., vomiting, stomachache, or rash), 19 patients without any hypersensitivity reaction and 16 healthy children never exposed before to PEGylated drugs, as controls. The presence of anti-PEG antibodies was determined with commercial ELISA tests for IgG and IgM and with a newly developed and characterized SPR assay, which allowed in the same sample simultaneous recognition of both the anti-PEG antibodies and the anti-ASP antibodies. These analyses allowed us to compare the cut-point determined with statistical approaches in the control subjects with the clinically significant cut-point, i.e., the threshold determined with a receiver operating characteristic (ROC) curve, which allows the prediction of hypersensitivity reactions with the best sensitivity and specificity values. Moreover, these analyses provide further data to elucidate the role of anti-PEG antibodies, determined with two methods, and of anti-ASP antibodies in the hypersensitivity reactions observed after administration of PEG-ASP.

## 2. Materials and Methods

### 2.1. Human Sera

We performed a post hoc analysis of 52 sera from 44 ALL patients diagnosed and treated with PEG-ASP in centers belonging to the AIEOP (Associazione Italiana di Ematologia e Oncologia Pediatrica) network, according to the AIEOP-BFM ALL 2009 (ClinicalTrials.gov identifier: NCT01117441) and AIEOP-BFM ALL 2017 (ClinicalTrials.gov identifier: NCT03643276) protocols. All trials were conducted in accordance with the Declaration of Helsinki and applicable national laws. The study protocol was approved by the institutional ethics committee, and appropriate written informed consent was obtained from the parents or legal guardians of all patients participating in the study trials.

The chemotherapy treatment consisted of intensive chemotherapy schedules subdivided into different phases (for further details please refer to the description available at the Clinical.Trials.gov website, NCT01117441 and NCT03643276). Twenty-seven sera were obtained from 25 patients who experienced clinically relevant reactions to the drug during any of the planned treatment phases including PEG-ASP doses. The classification of the reactions followed a two-step process. At the time of the event, the treating physician assigned an initial clinical diagnosis and classified each reaction as Grade 2 or higher according to Common Terminology Criteria for Adverse Events (CTCAE). Subsequently, all reactions were centrally reviewed by the coordinating center and reclassified as hypersensitivity reactions or allergic-like reactions according to the consensus definitions of the Ponte di Legno toxicity working group [28,29]: hypersensitivity reactions were defined as clinical allergic manifestations associated with a documented reduction in ASP activity, whereas allergic-like reactions were defined as clinical symptoms occurring without reduction in ASP activity. This central reclassification was performed independently of the anti-PEG/anti-ASP antibody results.

Notably, 7/25 patients (28%) reacted during the first dose of PEG-ASP, while the remainder occurred during subsequent infusions. For these patients, sera were obtained from blood samples taken 1 h after the event.

We also analyzed 25 sera from 19 ALL patients who did not experience clinically relevant reactions to the drug. In these cases, blood samples were taken 7 ± 2 days after the second PEG-ASP treatment in the re-induction phase.

It should be noted that although some patients contributed more than one sample to the analysis, these samples were taken at different time points during the treatment schedule (>1 month between blood collections) (Table S1). Thus, for the statistical analysis, these samples were considered independent each other.

Pre-existing anti-PEG antibodies were also evaluated in subjects never exposed before to PEGylated drugs. For this analysis, we used serum samples derived from laboratory testing performed for routine controls in 16 healthy children, obtained from the Laboratory of San Gerardo Hospital and classified as fully anonymized leftover material in accordance with institutional policies and ethical standards.

### 2.2. ASP Activity

ASP activity levels were determined using the aspartic acid β-hydroxamate (AHA, an analogue of Asn) test, using two calibration ranges of 5–100 IU/L and 100–1000 IU/L [30]. The method analyses the PEG-ASP catalytic activity in serum by detecting the amount of the hydrolyzed substrate AHA, quantified by photometric reading at 690 nm. The assay uses calibrators containing native enzyme preparation from *E. coli* (ASP, Medac GmbH, Wedel, Germany) reaching a lower limit of quantification (LLOQ) of 5 IU/L.

### 2.3. ELISA for Anti-PEG Antibodies (IgG and IgM)

Commercial ELISA kits for the detection of human anti-PEG IgG and human anti-PEG IgM (Life Diagnostics, Inc., West Chester, PA, USA) were used to analyze the sera from ALL patients following the manufacturer instructions.

These assays use, as the capture antigen, mono PEGylated BSA (20 kDa PEG chain). Different concentrations of anti-PEG IgG and IgM standards (100, 50, 25, 12.5, 6.25 and 3.125 U/mL) were incubated, alongside with sera samples diluted 100 (IgG) or 500-fold, in the microtiter wells on a plate shaker at 150 rpm/25 °C for 1 h. The wells were subsequently washed 5 times with 1× wash solution (400 µL/well). Then, 100 µL of horseradish peroxidase (HRP) conjugate was then added to the wells and incubated on a plate shaker at 150 rpm/25 °C for 45 min. The HRP conjugate binds to the anti-PEG molecules that are thus sandwiched between immobilized PEG and the detection antibody conjugate. Then, the plate was washed 5 times with 1× wash solution (400 µL/well) to remove unbound HRP-labelled antibodies, and 100 µL of 3,3′,5,5′-Tetramethylbenzidine (TMB) was added to the wells and incubated on a plate shaker at 150 rpm/25 °C for 20 min. In the presence of hydrogen peroxide, TMB is oxidized by HRP leading to the development of a blue color. Next, 100 µL of stop solution was added to each well, changing the color to yellow. Optical density was then measured at the spectrophotometer at 450 nm. The calibration curves were plotted using the absorbance values and concentrations of the standards. The absorbance values of the sera of ALL patients were interpolated to these standard curves to determine the concentration of anti-PEG antibodies.

### 2.4. Surface Plasmon Resonance (SPR) Assays for Anti-PEG and Anti-ASP Antibodies

For the present studies, a ProteOn XPR36 Protein Interaction Array system (Bio-Rad Laboratories, Inc., Hercules, CA, USA) was used. This system allows the immobilization of up to six ligands on parallel strips of the same sensor surface [31]. The microfluidic system is then rotated 90° so that up to six solutions can be flowed in parallel, simultaneously over the different immobilized ligands, enabling the analysis of up to 36 interactions in a single run and on a single chip.

Anti-PEG antibodies were recognized by immobilized methoxy–PEG—bovine serum albumin (PEG-BSA, PEG = 20 kDa, Life Diagnostics, Inc., West Chester, PA, USA), whereas ASP (Spectrila, Medac GmbH, Wedel, Germany) was immobilized to detect anti-ASP antibodies. A surface with immobilized BSA (Sigma-Aldrich Co. LLC, St. Louis, MO, USA) was always prepared in parallel and used as the reference surface.

The ligands (PEG-BSA, ASP and BSA) were immobilized by amine coupling on CMD200M chips (XanTec bioanalytics GmbH, Düsseldorf, Germany), functionalized with carboxylic acid groups. Before its use, the chips were initialized with glycerol 50% and then pre-conditioned with 0.5% SDS, 50 mM NaOH and 100 mM HCl.

For the immobilization, the chip surface was activated by flowing a solution made of 50 mM N-hydroxy succinimide (NHS) and 400 mM 1-ethyl-3-(3-dimethyl aminopropyl)-carbodiimide (EDC), for 5 min at 30 µL/min. Then, the ligands (PEG-BSA and ASP 30 µg/mL in acetate buffer 10 mM pH 4.0; BSA in acetate buffer 10 mM pH 4.5) were flowed for 2–5 min at 30 µL/min, followed by a further injection step with BSA alone to cover the whole surface. The remaining activated carboxyl groups were deactivated by flowing 1 M ethanolamine for 5 min at 30 µL/min.

After rotation of the fluidic system, sera containing the anti-PEG antibodies or not were injected in parallel surfaces on all the immobilized ligands. Initial characterization studies were carried out with control human sera spiked with different concentrations of commercial anti-PEG antibodies (anti-PEG IgG1 antibody from Acrobiosystem and Abcam, anti-PEG IgM antibody from Prodotti Gianni, Milano, Italy). Following studies aimed at evaluating the presence of anti-PEG antibodies in subjects who were never exposed to PEGylated drugs (“pre-existing” antibodies) and in patients treated with PEG-ASP.

Before the injection, sera samples were centrifuged at 10,000 rpm and 4 °C for 4 min and then diluted 30 times in a Tris-buffered saline (TBS) with 0.1% BSA and no Tween (the presence of BSA abolishes the non-specific binding of sera components to immobilized BSA, while Tween-20 might interfere with the measurements of anti-PEG antibodies due to its similarity to PEG [17,32]). Diluted sera flowed over immobilized ligands for 200–300 s (as indicated) at 30 µL/min, followed by a 10 min dissociation phase with running buffer only (TBS + 0.1% BSA). All these assays were done at 25 °C. The sensorgrams (time course of the SPR signal in resonance units, RU, where 1000 RU = 1 ng/mm^2^) were normalized to a baseline of zero. The signals in the surfaces immobilizing the ligands were corrected by subtracting the nonspecific response in the reference surface (BSA). In these experimental conditions, no binding of control serum was observed on immobilized BSA or PEG-BSA, and no binding of anti-PEG antibodies was observed on immobilized BSA [33].

Different regenerating solutions were also tested in order to re-use the same chip surface for the analysis of sera of different subjects in sequence, even after sera containing anti-PEG antibodies with a strong binding for immobilized PEG (i.e., very slow dissociation rates). To determine the best regenerating solutions, the injection of commercial anti-PEG antibodies was followed by the injection of different regenerating solutions, including the one described in Beeg et al., 2019 [26] (3.66 M MgCl_2_: 50 mM EDTA, 9:1) and different cocktails of the commercial stock regenerating solutions from XanTec BioAnalytics [34]. The best regeneration procedure, selected as described in the Results, was obtained by sequential injection of 3.66 M MgCl_2_:50 mM EDTA (9:1) and of a solution obtained mixing equal volumes of 20 mM EDTA and a solution made with equal volumes of DMSO, formamide, ethanol, acetonitrile and 1-butanol) and water.

### 2.5. Data Analysis

ROC analysis was carried out using the GraphPad Prism version 10.0.0 for Windows (GraphPad Software, Boston, MA, USA), using as binary classification the absence or presence of frank reduction of ASP activity, identified by ASP activities higher or lower than 500 IU/L. This is a conservative value, chosen on the basis of data showing that one hour after 2500 IU/m^2^ PEG-ASP, expected ASP activities range from 600 to 2000 IU/L [35], with a quite large interindividual variability. Moreover, our previous data [36] showed that, at day 7, the percentage of measures below 500 IU/L was 0.5% (2 out of 390 cases). Considering, in addition, that the activity has diminished in the first week, it is very unlikely to observe less than 500 IU/L in the first day in the absence of inactivation. This classification was in agreement with the clinical discrimination between hypersensitivity reactions and allergic-like reactions.

For ROC analysis, the measured ASP activities were normalized taking into account the amount of PEG-ASP effectively administered which, in many instances, was less than that planned due to the appearance of hypersensitivity reactions (Table S1).

The cut-point allowing the best sensitivity and specificity values was identified using the Youden index [37,38].

ELISA and SPR assays were also compared with a binomial test included in Prism (https://www.graphpad.com/guides/prism/latest/statistics/stat_how_to_mcnemars_test.htm, accessed on 31 August 2026).

## 3. Results

### 3.1. ASP Activity

Normalized ASP activity values < 500 IU/L, which confidently could suggest a reduction in drug activity (see Section 2), were found in 18 out of the 27 samples from 25 ALL children experiencing reactions to PEG-ASP, indicating a hypersensitivity with drug inactivation likely due to antibodies (Table S1).

Consistent with the previous pharmacokinetic data [35], ASP activities ≥ 1000 IU/L were measured in all the 25 serum samples collected 7 days after drug infusion from 19 ALL patients who did not experience hypersensitivity reactions (Table S1).

### 3.2. ELISA

Preliminary ELISA analysis was carried out on sera samples from 16 control children never exposed before to PEGylated drugs but possibly having pre-existing anti-PEG antibodies. The results are shown in Figure 1A. Different methods are proposed in the literature for the definition of the cutoff, e.g., indicating as “positives” the samples with values higher than the mean + 1.645 SD (the 95th percentile of the normal distribution [7,39]) or, more conservatively, the mean + 3SD (the 99.7th percentile of the normal distribution [14]). In this case, the former approach will result in cut-points of 6.78 and 37.25 U/mL for IgG and IgM, respectively, indicating two “positive” samples possibly containing pre-existing antibodies (for both IgG and IgM, Figure 1A,B), whereas the latter approach would indicate no pre-existing antibodies in any sample.

Figure 1C,D show the results obtained in the sera of ALL patients, in relation with the corresponding ASP activity measured in parallel. As regards IgG (Figure 1C), it visually appears that most of the samples with ASP activity > 500 IU/L have IgG levels ≤ 25 U/mL whereas, conversely, most samples with lower ASP activity have IgG levels higher than 30 U/mL. Figure 1E shows the corresponding ROC curve (see Section 2.5 for details), indicating an area under the curve of 0.92 (95% IC 0.84–1.00). The cut-point allowing the best sensitivity and specificity values (83.3% with 95% IC 60.8–94.2% and 91.2% with 95% IC 77.0–96.9%, respectively) was identified at 23.5 U/mL, notably higher than the cut-point determined with the less conservative statistical approach (i.e., 6.8 U/mL, with this cutoff, the specificity would drop to 68%). There were three samples in which anti-PEG IgGs levels were higher than the cut-point despite ASP activities > 500 UI/L (red in Figure 1C, possibly due to not neutralizing antibodies, in particular for the sample in which the ASP activity was 1653 UI/L), and three samples showing anti-PEG IgGs levels < cut-point despite the lack of significant ASP activity (green in Figure 1C). IgM did not appear to be clearly associated with drug activity (Figure 1D), since the area under the ROC curve was only 0.72 (Figure 1F).

### 3.3. Characterization of SPR-Based Assay for the Measurement of Anti-PEG Antibodies in Human Sera

For the characterization of the SPR method, control human sera were spiked with commercial anti-PEG antibodies and flowed over sensor chips pre-coated with PEG-BSA. A parallel surface of the same chip was always coated with BSA, as an internal reference.

Figure 2 shows the PEG-specific binding obtained with 30-fold diluted sera spiked with different concentrations of commercial anti-PEG antibodies, either IgM or IgG1, injected sequentially from the lower to the higher concentration (without regeneration). The results indicated a concentration-dependent SPR signal, with a signal clearly detectable already at the lower concentration tested (1 nM, final concentration), and estimated limits of detection (LOD) of 6.1 and 3.5 µg/mL of undiluted sera for IgM and IgG1 (Abcam). These values were in the same order of magnitude of the clinically relevant anti-infliximab antibodies we investigated previously [26].

These SPR data also highlighted a marked difference in the binding properties of the two commercial anti-PEG IgG1 tested: the Acrobiosystem IgG1 is characterized by a dissociation rate much faster than that of Abcam IgG1 but, also, by a much faster association rate (a marked binding signal is already observed at the lowest concentration despite the fast dissociation rate). It is likely that the two IgG1 antibodies have been selected by the vendors for a “high affinity” for PEG (low equilibrium dissociation rate or K_D_), but SPR highlights the important differences in the underlying association and dissociation binding constants.

Since the SPR assay aims to detect anti-PEG antibodies in sera from different subjects, and since some antibodies might bind in a very strong manner (i.e., with a very slow dissociation rate), it was necessary to identify appropriate regeneration procedures suitable to completely remove the bound antibody from the antigen immobilized on the chip, without affecting the binding properties of the latter for testing subsequent sera samples. These requisites were satisfied by a regenerating solution (see Section 2) that allows a removal of 99% of the bound analyte.

### 3.4. SPR Analysis of Sera from Control Children and ALL Children in Treatment with PEG-ASP

For SPR studies, the sera flowed simultaneously on immobilized ASP, PEG-BSA or BSA as the reference surface, in order to detect in the same sample, the presence of anti-ASP and/or anti-PEG antibodies.

No ASP- or PEG-dependent SPR signal was observed in any of the sera from the 16 control children (Figure 3A).

Figure 3B shows that measurable (i.e., higher than the instrument noise) PEG-specific SPR signals were found in 18 out of the 27 sera samples taken from ALL children with hypersensitivity to PEG-ASP. Figure 3C shows a representative sensorgram to highlight the heterogeneity of a patient’s anti-PEG antibodies, as indicated by the presence of multiple dissociation rates (i.e., part of the bound antibodies dissociates very rapidly, where others remain strongly bound to the immobilized PEG at the end of the dissociation phase).

Figure 3B shows the anti-PEG antibodies in relation with the corresponding ASP activity. Applying ROC analysis, an area under the curve of 0.81 (95% IC = 0.67–0.95) was estimated, while the cut-point allowing the best sensitivity/specificity values (66.7% and 94.1% respectively) was identified at 46.5 RU. Figure 3B shows that there are two samples in which we measured anti-PEG antibodies (> cut point) even in the presence of normal ASP activity (red in Figure 3B), possibly due to not-neutralizing antibodies or to other PEG-binding components of the sera, and six samples showing an SPR signal < 46 RU despite the lack of significant ASP activity (green in Figure 3B). Two of the latter (empty green circle in Figure 3B), however, are the ones found to have significant anti-ASP SPR signals (>50 RU) (Table S1). Thus, the simultaneous SPR measurement of both anti-drug and anti-PEG antibodies allows a reduction in the number of “false negatives” to four. Considering both anti-PEG and anti-ASP antibodies for ROC analysis (Figure 3D), the area under the curve increased to 0.84 (95% IC = 0.71–0.97), with sensitivity and specificity values of 77.9% (95% IC = 54.8–91.0) and 94.1% (95% IC = 80.9–98.9) respectively. These values were not statistically different from those obtained with ELISA, as assessed comparing the two assays with a binomial test (*p* = 0.625).

Significant levels of anti-ASP antibodies (>50 RU) were also detected in four samples together with anti-PEG antibodies (Table S1).

## 4. Discussion

Although PEG has low immunogenic potential, and the PEGylation of biological drugs effectively reduces their immunogenicity [6], hypersensitivity reactions to PEGylated drugs have been observed, and anti-PEG antibodies have been indicated as a main cause [7,10,11,12]. An important, still unresolved, question regards the possibility of reliably exploiting the measurement of anti-PEG antibodies to guide appropriate therapeutic decisions, e.g., switching to a different product in case of a hypersensitivity reaction to a PEGylated form or predicting patients at risk of clinical allergy due to pre-existing anti-PEG antibodies. This requires the availability of robust methods able to recognize clinically relevant levels of anti-PEG antibodies with adequate sensitivity and specificity. To this end, in the present study, we used two analytical assays to measure anti-PEG antibodies in ALL children who showed hypersensitivity reactions to PEG-ASP, either clinical allergy associated with ASP inactivation (possibly related to the presence of anti-PEG or anti-ASP antibodies), or allergic-like reactions with no major reduction of drug activity; the same evaluations were performed in a subset of healthy children used as controls. The availability of sera samples obtained from the two groups of ALL children allowed us to determine the sensitivity and the specificity of the methods with clinically relevant cut-points, i.e., determined with ROC analysis using ASP activity for the binary classification.

Commercial ELISA kits were used to measure anti-PEG IgG and IgM. The use of a cutoff estimated by ROC analysis showed that anti-PEG IgGs are predictive of drug inactivation with good sensitivity and specificity: they are present in 15 out of the 18 samples with significantly impaired ASP activity (sensitivity = 83.3%) and in only 3 out of 34 samples with sufficient ASP activity (specificity = 91.1%).

Notably, much lower specificity (68%) would have been observed by using cut-points defined with standard statistical approaches based on the results obtained in controls. These data further strengthen the importance of a correct identification of cut-points, often overlooked in the literature, resulting in the underestimation of potentially good predictors.

Our results also show that the presence of anti-PEG IgM does not predict drug inactivation with sufficient specificity and sensitivity.

To further corroborate and extend the ELISA results, we developed and characterized an SPR-based assay, which allows simultaneously recognizing anti-PEG and anti-ASP antibodies (the latter being potentially important to explain a lack of ASP activity despite the lack of anti-PEG antibodies). This was based on our previous work in which we validated and exploited SPR to simultaneously detect infliximab and anti-infliximab antibodies [26], showing, in particular, that the real-time measurements possible with SPR, at variance with the multiple incubations and washing steps required by ELISA, might allow more reliable detection of patient’s low-affinity antibodies [27].

The novel SPR-based assay recognized anti-PEG antibodies in a specific and concentration-dependent manner. Although an SPR-based method has been recently published [24], the procedure was quite laborious, in particular for the immobilization procedures on custom-built SPR sensor surfaces. The protocol adopted here employs commercial chips and the immobilization of PEG-BSA with a classical amine-coupling approach and can be easily applied on most SPR platforms.

Measurable SPR signals, specific for PEGylated surface, were found with 18 out of the 52 sera obtained from children treated with PEG-ASP, whereas no SPR signal was observed with any of the control sera, indicating the lack of measurable levels of pre-existing antibodies in the pediatric population considered in the present study. These data are consistent with the literature data [7], suggesting the possibility that the development of the so-called pre-existing anti-PEG antibodies is age-dependent and due to continuous exposure to PEG-containing everyday products.

One of the advantages of the SPR real-time measurements is the possibility to determine the association and dissociation binding rates of the antibodies, which cannot be evaluated by other methods measuring the binding at equilibrium, such as ELISA. This was evident with the two commercial anti-PEG IgG1 antibodies used for the method characterization, which showed very different association/dissociation binding rates. Similar differences might be also expected for the anti-PEG antibodies produced in humans exposed to PEGylated products [6]. Consistently, the SPR analysis of sera samples from ALL patients unveiled, even in the same sample (e.g., see Figure 3C), the presence of heterogeneous populations of anti-PEG antibodies, characterized by different dissociation rates: some of them remain bound in a very strong manner, whereas others dissociate very rapidly. The latter could be potentially lost during the washes of the ELISA assay, as we previously demonstrated for anti-infliximab antibodies [27], and could explain why some samples with very high SPR signals did not show comparatively high ELISA signals. Despite this, however, very similar results were obtained with ELISA and SPR, both predicting drug inactivation with good sensitivity and specificity (>79% and >91%, respectively). In this case, no statistically significant differences between the two assays were observed.

For SPR too, the clinically-relevant cut-point (46 RU, as determined by ROC curves) was higher than the cut-point that could have been determined by statistical approaches (taking into account that experimental noise is about 5 RU and that no sample in the control group showed a binding higher than 5 RU). These findings highlight the following: (i) the SPR assay is sufficiently sensitive for the detection of clinically relevant anti-PEG antibodies, and (ii) in this case, the higher sensitivity of ELISA in comparison with SPR does not represent a relevant advantage. ELISA kits allow, however, identification of the type of antibody (e.g., IgG or IgM), whereas with SPR, this is possible only for the antibodies that do not dissociate from the immobilized PEG. On the other hand, SPR allows simultaneous measurement, in the same sample and very rapidly, of both the anti-PEG and the anti-ASP (anti-drug) antibodies, allowing a comprehensive identification of correct specificity and sensitivity values. In the present study, for example, we found two samples with low ASP activity that were negative for anti-PEG antibodies (in both ELISA and SPR) but positive for anti-ASP antibodies. 

Overall, the results of our study indicate that 18 out of 27 samples collected in children with hypersensitivity reactions (67%) were associated with a clinical allergy with impaired ASP activity. These reactions could be predicted by anti-drug antibody measurements by both assays with good sensitivity and specificity (>78 and >91%, respectively). In a few of these 18 samples (three for ELISA and four for SPR), the impairment of ASP activity was not associated with measurable anti-drug antibodies, indicating that other reasons or antibodies not detectable with the used assays explain the ASP inactivation. Nine samples were from patients with allergic-like reactions without impairment of ASP activity, and only two of them showed the presence of anti-PEG antibodies (likely not-neutralizing antibodies).

A limitation of the present study is the relatively small number of patients considered, due to the rarity of the childhood ALL and the relatively small incidence of allergic reactions (7.7% on average, [9]). Although we are confident that the reported findings have a solid ground, we acknowledge that the identified cut-points remain exploratory, and external validation is necessary before clinical implementation. The limited sample size also precluded multivariate analysis. 

In summary, the results of our study are important not only from the methodological and laboratory point of view but also from the clinical point of view, since the application of such testing, once the results will be confirmed on a larger scale, could effectively complement the usually available information related to asparaginase activity levels to make the PEG-asparaginase treatment even more rational and evidence-based.

## Figures and Tables

**Figure 1:**
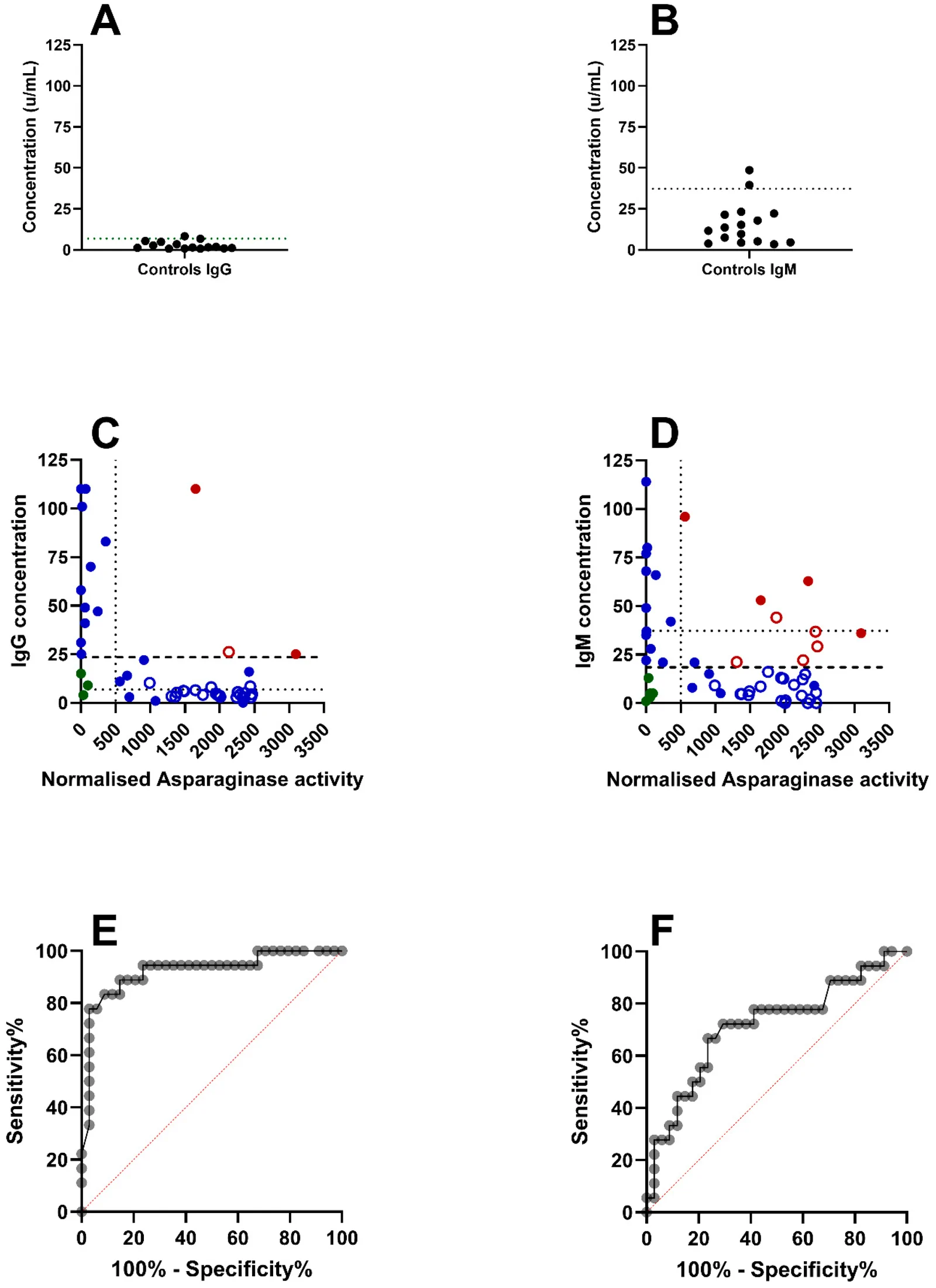
Results of the ELISA assay for anti-PEG IgG and IgM antibodies in sera. Panels (**A**,**B**) show the results obtained in sera from 16 pediatric subjects never exposed to PEGylated drugs ((**A**): IgG; (**B**): IgM). Panels (**C**,**D**) show the results obtained from 52 sera from pediatric patients treated with PEG-ASP ((**C**): IgG; (**D**): IgM, in relation with ASP activity measured in the same samples). The horizontal dotted lines indicate the technical cut-points estimated from control subjects as mean + 1.645 SD. The bold horizontal dashed line in panel C indicates the cut-point with the best sensitivity/specificity values as determined by the ROC analysis. Blue points indicate results from sera in which the absence/presence of antibodies is consistent with the ASP activity: filled ones were from samples taken 1 h after treatment in ALL patients with hypersensitivity reactions, while empty ones were from samples taken 7 ± 2 days after treatment in ALL patients with no reactions; red points indicate the presence of antibodies despite presence of activity, while green points indicate the lack of antibodies despite the lack of activity. (**E**) ROC curve of IgG, showing an area under the curve of 0.92 (95% IC = 0.84–1.0). (**F**) ROC curve of IgM, showing an area under the curve of 0.72 (95% IC = 0.56–0.87).

**Figure 2:**
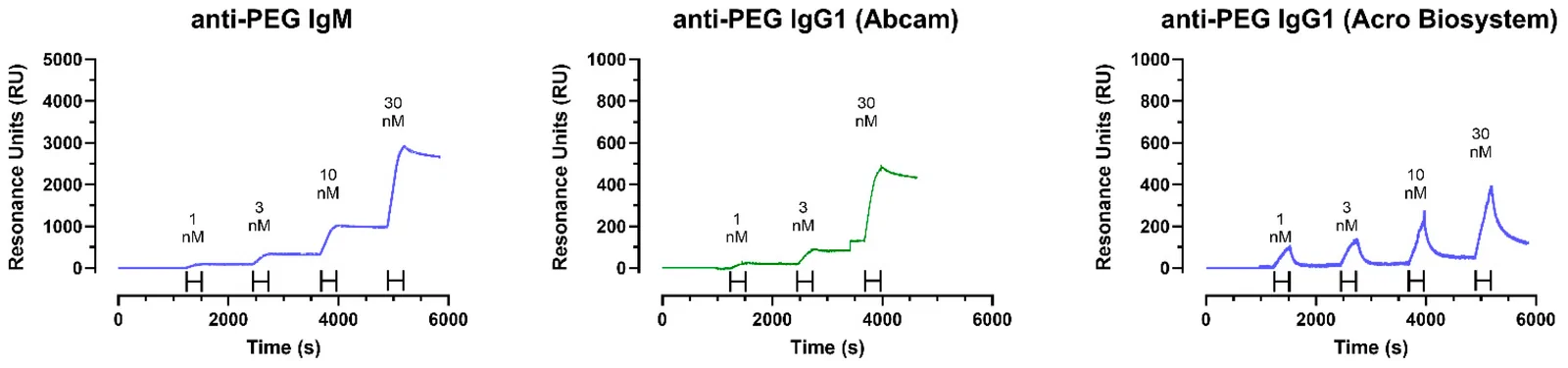
SPR binding signals with commercial anti-PEG antibodies. Sensorgrams (i.e., time course of the SPR signal in resonance units) obtained with sequential injections of increasing concentrations of commercial anti-PEG antibodies. The antibodies were injected for three min, as indicated, followed by 10 min of dissociation phase; there was no regeneration between subsequential injections. These sensorgrams show the specific PEG-dependent binding, obtained as the difference between the signal measured on PEG-BSA surface and the signal measured on BSA surface.

**Figure 3:**
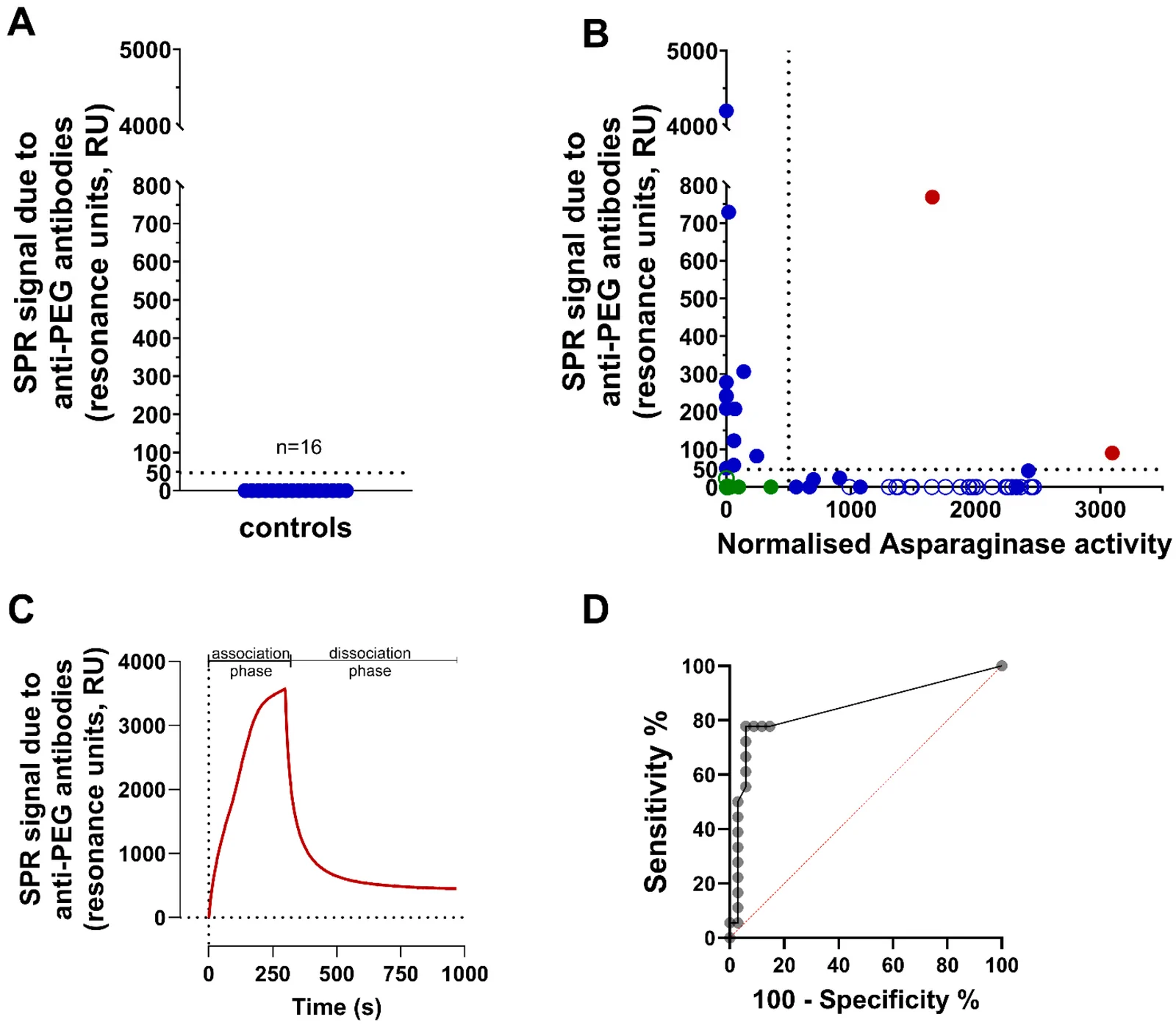
Results of SPR analysis for anti-PEG antibodies in sera. Panel (**A**) shows the SPR results obtained with sera from 16 pediatric subjects never exposed to PEGylated drugs. Panel (**B**) shows the SPR results obtained with 52 sera from pediatric patients treated with PEG-ASP, reported in relation with ASP activity measured in the same samples (normalized on the dose actually administered). Horizontal dotted lines indicate the cut-point values, which for anti-PEG antibodies is the SPR signal allowing the best specificity/sensitivity values (47 RU, determined from the ROC curve in panel (**D**)). Blue points indicate results from sera in which the absence/presence of antibodies are consistent with the ASP activity: filled ones were from samples taken 1 h after treatment in ALL patients with hypersensitivity reactions, while empty ones were from samples taken 7 ± 2 days after treatment in ALL patients with no reactions; red points indicate the presence of antibodies despite the presence of activity, while green points indicate the lack of antibodies despite the lack of activity (empty green circles were from samples containing anti-ASP antibodies). (**C**) Representative sensorgram showing the specific binding to immobilized PEG after injection of a serum sample from an ALL patient. The sensorgram highlights the presence of both quickly dissociating anti-PEG antibodies and anti-PEG antibodies that remained bound at the end of the dissociation phase. (**D**) ROC analysis showed an area under the curve of 0.844 (95% IC = 0.714–0.973).

## Data Availability

The original contributions presented in this study are included in the article/Supplementary Materials. Further inquiries can be directed to the corresponding authors.

## Supplementary Materials

The following supporting information can be downloaded at https://www.mdpi.com/article/10.3390/pharmaceutics18101196/s1, Table S1: Individual patient data: PEG-ASP activity and anti-drug antibody measurements.

## Abbreviations

The following abbreviations are used in this manuscript:

ALL	Acute Lymphoblastic Leukemia
ASP	L-asparaginase
PEG	Polyethylene Glycol
ELISA	Enzyme-Linked Immunosorbent Assay
SPR	Surface Plasmon Resonance
ROC	Receiver Operating Characteristic
AIEOP	Associazione Italiana di Ematologia e Oncologia Pediatrica
CTCAE	Common Terminology Criteria for Adverse Events
AHA	Aspartic Acid β-hydroxamate
BSA	Bovine Serum Albumin
HRP	Horseradish Peroxidase
TMB	3,3′,5,5′-Tetramethylbenzidine
NHS	N-hydroxy succinimide
EDC	1-ethyl-3-(3-dimethyl aminopropyl)-carbodiimide
TBS	Tris-buffered Saline
RU	Resonance Units
