# Supplementary material for: Evaluating the Role of Anti-PEG Antibodies in Hypersensitivity to PEG-Asparaginase Through Clinically Relevant Cut-Points Defined by ELISA and Surface Plasmon Resonance
Source: Pharmaceutics. 2026 Sep 22;18(10):1196. doi: 10.3390/pharmaceutics18101196 (PMC13708339; doi:10.3390/pharmaceutics18101196)
Supplement: Supplementary file 1 [file pharmaceutics-18-01196-s001.zip › pharmaceutics-4473590-supplementary.pdf]

Table S1: *Individual patient data: PEG-asparaginase activity and anti-drug antibody measurements.*

patient #	PEG-ASP Protocol phase	PEG-ASP reaction	measured ASP activity U/L	Administered dose (%)	normalized ASP activity U/L	IgG ELISA (U/ml)	IgM ELISA (U/mL)	anti-PEG SPR (RU)	anti-ASP SPR (RU)
1	2_III	no reaction	**2328**	100%	2328	3	0	0	0
2	II	no reaction	**1944**	100%	1944	5	13	0	0
3	HR1	no reaction	**1362**	100%	1362	3	5	0	0
3	1_III	no reaction	**2450**	100%	2450	3	0	0	0
3	3_III	no reaction	**2007**	100%	2007	3	1	0	0
4	1_III	no reaction	**2002**	100%	2002	4	0	0	0
4	2_III	no reaction	**2241**	100%	2241	3	4	0	0
5	HR1	no reaction	**1382**	100%	1382	5	5	0	0
5	HR3	no reaction	**1489**	100%	1489	6	6	0	0
5	1_III	no reaction	**1950**	100%	1950	5	1	0	0
6	HR3	no reaction	**1478**	100%	1478	6	4	0	0
6	HR2	no reaction	**989**	100%	989	10	9	0	0
7	II	no reaction	**2261**	100%	2261	6	22	0	0
8	II	no reaction	**2466**	100%	2466	4	29	0	0
9	II	no reaction	**1305**	100%	1305	3	21	0	0
10	II	no reaction	**2132**	100%	2132	26	9	0	0
11	II	no reaction	**2440**	100%	2440	8	37	0	0
12	II	no reaction	**2363**	100%	2363	5	2	0	0
13	HR2	no reaction	**1649**	100%	1649	6	9	0	0
14	II	no reaction	**2291**	100%	2291	4	15	0	0
15	II	no reaction	**2448**	100%	2448	4	5	0	0
16	II	no reaction	**2255**	100%	2255	5	12	0	0
17	II	no reaction	**1977**	100%	1977	4	13	0	0
18	II	no reaction	**1758**	100%	1758	4	16	0	0
19	II	no reaction	**1878**	100%	1878	8	44	0	0
20	ConsBext	Hypersensitivity reaction	**<LOQ**	12,5%	0	215	68	242	128
21	IA	Hypersensitivity reaction	**<LOQ**	1%	0	197	114	240	70
22	ConsBext	Hypersensitivity reaction	**<LOQ**	100%	0	58	22	50	27
23	IA	Allergic like reaction	**140**	25%	560	11	96	0	27
24	ConsBext	Hypersensitivity reaction	**< LOQ**	33%	0	142	22	0	30
25	IA	Hypersensitivity reaction	**358**	100%	358	83	42	0	27
26	ConsBext	Hypersensitivity reaction	**8**	100%	8	25	37	0	73
27	ConsBext	Hypersensitivity reaction	**<LOQ**	100%	0	215	49	208	101
28	IA	Allergic like reaction	**397**	17%	2335	0	63	0	47
29	ConsBext	Allergic like reaction	**628**	38%	1653	110	53	769	0
29	HR1	Hypersensitivity reaction	**11**	16%	69	110	28	207	0
30	IA	Hypersensitivity reaction	**9**	48,6%	19	101	80	729	1001
31	HR2	Hypersensitivity reaction	**35**	25%	140	70	66	306	0
32	IB	Hypersensitivity reaction	**<LOQ**	100%	0	15	35	133	0
33	HR2	Hypersensitivity reaction	**<LOQ**	50%	0	110	77	4200	0
34	II	Hypersensitivity reaction	**7**	11,4%	61	49	5	123	0
35	IA	Allergic like reaction	**2423**	100%	2423	16	9	43	33
35	ConsBext	Hypersensitivity reaction	**59**	100%	59	41	3	58	13
36	II	Allergic like reaction	**<100**	11%	909	22	15	24	0
37	IA	Hypersensitivity reaction	**122**	50%	244	47	21	82	0
38	HR1	Hypersensitivity reaction	**<LOQ**	100%	0	31	1	23	90
39	IA	Allergic like reaction	**214**	30,6%	699	3	21	20	45
40	IA	Allergic like reaction	**>100**	15%	667	14	8	0	0
41	ConsBext	Hypersensitivity reaction	**> 100**	100%	100	9	5	0	0
42	IA	Hypersensitivity reaction	**36**	100%	36	4	13	0	0
43	IA	Allergic like reaction	**1075**	100%	1075	1	5	0	0
44	IA	Allergic like reaction	**743**	24%	3096	25	36	90	30

Abbreviations: ASP, asparaginase; ELISA, enzyme-linked immunosorbent assay; HR1/HR2/HR3, high-risk block 1/2/3; IA, induction IA; IB, induction IB; ConsB ext, consolidation B extended; II, protocol II; 1_III/2_III/3_III, first/second/third protocol III; LOQ, limit of quantification; PEG, polyethylene glycol; RU, resonance units; SPR, surface plasmon resonance.
